# Screening for Pandemic (H1N1) 2009 Virus among Hospital Staff, Spain

**DOI:** 10.3201/eid1706.100577

**Published:** 2011-06

**Authors:** Julián Olalla, Miguel Marcos, Fernando Fernández, Jaouad Oulkadi, Natalia Montiel, Alfonso del Arco, Víctor Fuentes, Javier de la Torre, José Luis Prada, Javier García-Alegría

**Affiliations:** Author affiliation: Hospital Costa del Sol, Marbella, Spain

**Keywords:** viruses, influenza, health care workers, pandemic (H1N1) 2009, pandemic, screening, Spain, letter

**To the Editor:** After the emergence of pandemic (H1N1) 2009 virus, measures for its control were taken quickly (e.g., isolation of affected patients and use of gowns, gloves, and N95 respirators) when a clinical suspicion of pandemic influenza was established (*1*). One population group frequently exposed to this virus is health care staff. These circumstances prompted us to implement a screening program for the pandemic (H1N1) 2009 virus among personnel working at our hospital in Marbella, Spain.

Costa del Sol Hospital is a 250-bed, second-level center located on the Mediterranean coast. A proposal was made to staff working in the emergency and internal medicine areas that nasal and pharyngeal samples to identify the virus by real-time PCR should be taken weekly over 12 consecutive weeks, from the third week of September 2009 to the third week of December. In addition to providing samples, each worker would be asked to complete a health-status questionnaire regarding his or her vaccination record and the presence of signs or symptoms. Signs and symptoms to be reported in the questionnaires included fever, runny nose, painful swallowing, coughing, sore throat, diarrhea, vomiting, headaches, muscle pains, and general indisposition; 1 question also asked whether, during the previous week, a confirmed diagnosis of influenza with a positive PCR for pandemic (H1N1) 2009 virus had been made in the respondent’s household.

At the outset, 60 members of the hospital staff volunteered to participate. Those who missed >4 sample tests, or >2 consecutive ones, were considered to have abandoned the study. Of the 36 staff members who completed the study, 27 were women (75%). The participants’ average age was 37 years (CI 95%: 34.8–39.4). Sixteen were doctors, 16 were nurses, 2 were nursing auxiliary staff, and 2 were hospital orderlies. During the monitoring period, 5 (13%) subjects exhibited coughing, 7 (20%) had runny noses, 3 (8%) experienced painful swallowing, 6 (16%) had headaches, and 1 (2%) felt generally unwell. Nearly 75% stated they washed their hands with antiseptic lotion >20×/d. Three workers were vaccinated against seasonal and pandemic influenza, while only 1 was vaccinated against pandemic (H1N1) 2009 alone. None took oseltamivir. Five positive samples were identified (13.8% of the study population) being obtained from four doctors and one nurse, all women. The 4 doctors had signs and symptoms for 24–48 hours consisting of fever, general indisposition, and coughing; none of the 4 required hospitalization. The nurse was a woman 26 years of age with no influenza symptoms and with a positive PCR result on week 5. None of these 5 workers had received any influenza vaccination.

Three workers reported that a diagnosis of pandemic (H1N1) 2009 influenza had been made with respect to a member of their household, but none of the workers had a positive PCR result. The distribution of positive PCR results in our hospital during the study is shown in the Figure.

**Figure:**
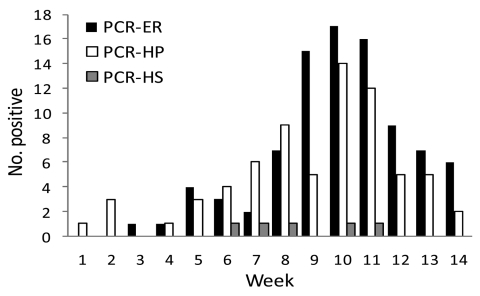
Number of PCR-confirmed cases of pandemic (H1N1) 2009 virus infection in the emergency department (PCR-ER), hospitalized patients (PCR-HP), and participants (PCR-HS) in a study of screening for pandemic (H1N1) 2009 virus among health care workers, Spain, September–December 2009.

It had previously been hypothesized that the incidence of asymptomatic cases would be higher than the incidence of symptomatic cases (*2*) overall in persons with high exposure (*3*). However, among the study population, only 1 person with positive PCR results was asymptomatic.

Health care workers may have been exposed in a gradual manner from the beginning of the outbreak to a few symptomatic forms, which would explain why so few of them were actually affected. Of the workers in the emergency department who were not part of the study, none were diagnosed with pandemic (H1N1) 2009 during the study period.

Our study began during the week in September 2009 in which the overall rate of incidence of pandemic (H1N1) 2009 in Spain reached 77.8 cases per 100,000 inhabitants (*4*), a level that was above the threshold established for the previous influenza season, and ended during the week in which influenza activity fell below this threshold level (*5*). Therefore, the study spanned the full cycle of the epidemic. The national peak, with an overall rate of incidence of 372.7 cases per 100,000 inhabitants, occurred in week 10 of our study.

This series included 1 asymptomatic carrier. We do not know if that finding could reflect a false-positive test or a low-virulence viral presence.

Notably, among the population of health care workers taking part in the study, only 4 (11%) had been vaccinated against the novel form of the influenza A virus, and none of them had positive PCR results for pandemic (H1N1) 2009 virus. On the other hand, 5 (15%) of workers not vaccinated had a positive PCR result. This finding suggests that, despite the climate of uncertainty concerning the evolution of the influenza outbreak, hospital workers had a greater fear of possible side effects of the vaccine than of the disease itself.
